# Predicting and retrodicting intelligence between childhood and old age in the 6-Day Sample of the Scottish Mental Survey 1947

**DOI:** 10.1016/j.intell.2015.02.002

**Published:** 2015

**Authors:** Ian J. Deary, Caroline E. Brett

**Affiliations:** Centre for Cognitive Ageing and Cognitive Epidemiology, Department of Psychology, University of Edinburgh, United Kingdom

**Keywords:** Intelligence, Ageing, IQ, Scottish Mental Survey, Longitudinal

## Abstract

In studies of cognitive ageing it is useful and important to know how stable are the individual differences in cognitive ability from childhood to older age, and also to be able to estimate (retrodict) prior cognitive ability differences from those in older age. Here we contribute to these aims with new data from a follow-up study of the 6-Day Sample of the Scottish Mental Survey of 1947 (original N = 1208). The sample had cognitive, educational, social, and occupational data collected almost annually from age 11 to 27 years. Whereas previous long-term follow-up studies of the Scottish mental surveys are based upon group-administered cognitive tests at a mean age of 11 years, the present sample each had an individually-administered revised Binet test. We traced them for vital status in older age, and some agreed to take several mental tests at age 77 years (N = 131). The National Adult Reading Test at age 77 correlated .72 with the Terman–Merrill revision of the Binet Test at age 11. Adding the Moray House Test No. 12 score from age 11 and educational information took the multiple R to .81 between youth and older age. The equivalent multiple R for fluid general intelligence was .57. When the NART from age 77 was the independent variable (retrodictor) along with educational attainment, the multiple R with the Terman–Merrill IQ at age 11 was .75. No previous studies of the stability of intelligence from childhood to old age, or of the power of the NART to retrodict prior intelligence, have had individually-administered IQ data from youth. About two-thirds, at least, of the variation in verbal ability in old age can be captured by cognitive and educational information from youth. Non-verbal ability is less well predicted. A short test of pronunciation—the NART—and brief educational information can capture well over half of the variation in IQ scores obtained 66 years earlier.

## Highlights

• Results are presented on a new follow-up sample of the Scottish Mental Survey of 1947.
• Terman–Merrill Stanford–Binet intelligence test scores at age 11 years correlated .72 with verbal intelligence at age 77.
• Adding another test from age 11 and educational took the multiple R to .81.
• We also attempted to estimate prior ability from verbal ability scores in older age.
• National Adult Reading Test scores at age 77 and educational had a multiple R of .75 with IQ scores at age 11.

## 1 Introduction

When cognitive data are available from the same individuals in childhood and older age, two types of investigation may be undertaken. First, one can ask how strongly childhood intelligence differences predict intelligence differences in older age, and which variables account for additional variance. This is important in order to understand which factors contribute to people's differences in cognitive ageing. Second, one can ask how strongly vocabulary-type tests—that are often used to estimate peak prior intelligence in older people—estimate (retrodict) measured intelligence from youth. This is important in validating estimates of prior, or premorbid, intelligence, which are valuable in applied and basic work with people who have pathological cognitive decline, such as is found in dementia. The present study does both, using data from the newly-revived 6-Day Sample of the Scottish Mental Survey 1947; an historical summary of the 6-Day Sample is given by Deary, Whalley, and Starr (2009, chapter 1), and the permissions required for the reviving of the study are described by Brett and Deary (2014).

With regard to predicting intelligence differences in middle and older age from those in youth, there are several studies that have administered the same intelligence-type tests at those far-apart points in the life course (Deary, 2014; Gow et al., 2011; Owens, 1966; Plassman et al., 1995; Schwartzman, Gold, Andres, Arbuckle, & Chaikelson, 1987). These studies are summarised and described in more detail by Deary (2014). An approximate summary would be that about half or less of the variance is stable between age 11 years and about age 70, i.e. the test–retest correlations are about or slightly less than .7. Test–retest correlations are higher between youth and middle age, and lower between childhood and age 90, though still about or greater than .5 (Deary, 2014). There is a search for the factors that contribute to the residual non-stable variance, because it is of considerable interest to discover any modifiable determinants of healthy cognitive ageing. There is evidence that, for example, not having the *APOE* e4 allele, not smoking, and being healthy (not having cardiovascular disease or diabetes, for example) and physically fitter and more active might contribute small amounts to healthier cognitive ageing (Deary et al., 2009; Plassman, Williams, Burke, Holsinger, & Benjamin, 2010). The generally-held view is that there are contributions from all stages of the life course to cognitive differences (Foresight Mental Capital & Wellbeing Project, 2008). Here, we focus on social and educational factors from youth, and we test whether such variables can add to the predictive validity of measured intelligence from youth in explaining cognitive differences in older age.

In a previous report, using a different follow-up sample of the Scottish Mental Survey 1947, we found that education (years of full-time education) contributed some variance in addition to prior cognitive ability in accounting for intelligence differences in older age (Ritchie, Bates, Der, Starr, & Deary, 2013). This was done using only a single, group-based intelligence test in youth. Moreover, the other previous long-term follow-up studies of the stability of intelligence differences based on the Scottish mental surveys (summarised by Deary, 2014) have all used data from the group-based intelligence test (the Moray House Test No. 12) that was administered at age 11 years. The present study is based on a new follow-up study of the 6-Day Sample of the Scottish Mental Survey of 1947. It has the advantage of the sample's having two broad and well-validated tests from age 11—one of which is an individually-administered revised Binet test and is broader in content. A second advantage of this sample is that there are detailed educational and social background and occupational data that were gathered almost yearly from age 11 to age 27, between 1947 and 1963. We have now administered several tests covering important domains of cognitive ability at a mean age of 77 years.

With regard to retrodicting prior intelligence differences from information gathered in middle and older age, and in states of cognitive decline, the tests that are used are based partly on the consistent finding that vocabulary-type cognitive tests hold better in older ages than do tests that require more active, on-the-spot thinking (Park & Bischof, 2013; Salthouse, 2004). This type of ‘hold’ refers to the fact that mean levels in vocabulary tests do not decline in older age the way that test scores of other cognitive domains—such as non-verbal reasoning, processing speed, and some aspects of memory—decline. This is necessary but not sufficient for vocabulary tests to be useful indicators of prior cognitive ability. There are two types of ‘hold’ with respect to the longitudinal stability of cognitive test scores as follows: the stability of mean levels and the stability of individual differences (Deary, 2014). Given the former, the latter does not necessarily follow. However, verbal tests also show stronger ‘hold’ when it comes to stability of individual differences. For example, in a 40-year follow-up study of 260 male Canadian World War II veterans who had taken the Revised Examination M Test of intelligence at conscription and again in older age, the correlations between youthful and older-age verbal and non-verbal scores were .82 and .54, respectively (Schwartzman et al., 1987). Similarly, in a follow-up of 96 freshmen tested on the Army Alpha in 1919 and again in 1961, the youthful versus older-age correlation for the reasoning component was .58, and the correlation for the verbal component was .73. Therefore, verbal tests show higher stability of both mean levels and individual differences.

The concept of estimating prior, or premorbid, intelligence was realised with the development of the National Adult Reading Test (Nelson & Willison, 1991), and subsequent similar tests such as the Wechsler Test of Adult Reading and the Test of Premorbid Functioning. These tests require the participants to pronounce words that are irregular in their grapheme-phoneme associations and/or stress. The idea is that one is unlikely to work out how to pronounce such words if one does not already know how they sound. To date, the NART and WTAR have been retrospectively validated in people with and without dementia (Crawford, Deary, Starr, & Whalley, 2001; Dykiert & Deary, 2013; McGurn et al., 2004); there are correlations between .6 and .7 between NART and WTAR scores in older age and group-administered intelligence test scores obtained at age 11 years. In the present study we focus on social and educational factors from youth, and we test whether such variables can add to the retrospective validity of the NART in estimating prior intelligence as was measured using both individually-administered and group-administered tests.

## 2 Method

### 2.1 Participants

**Fig. 1:**
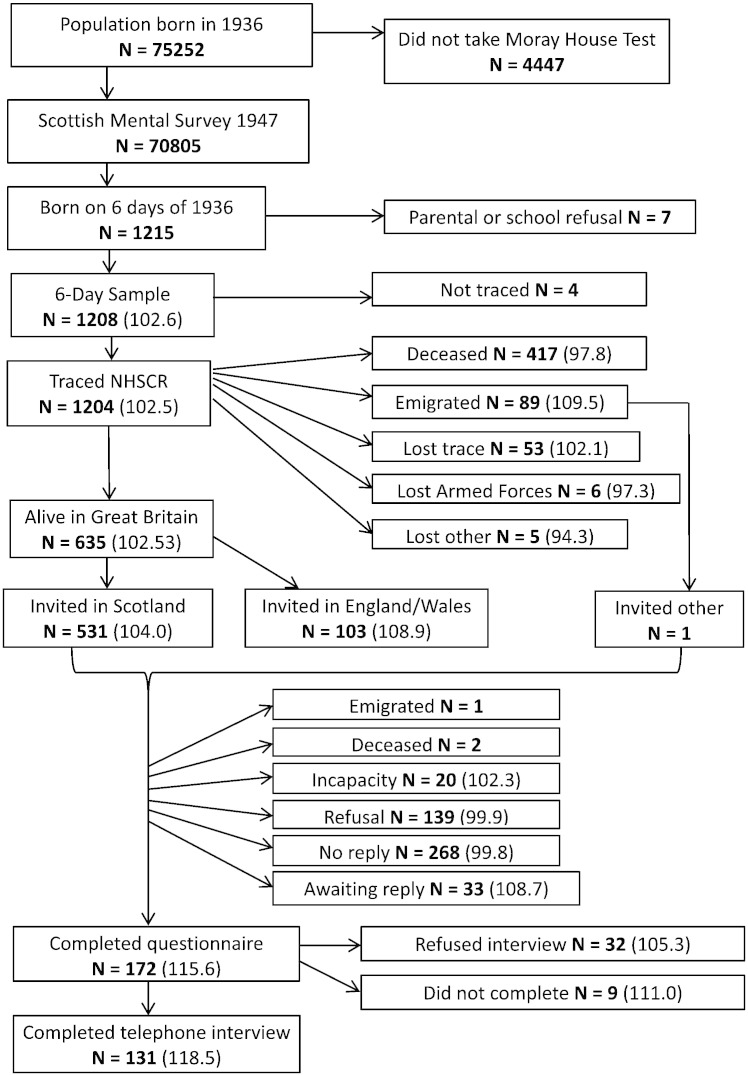
Diagram to show the relationship of the sample (N = 131) tested in the present study to the whole 1936-born Scottish population, the Scottish Mental Survey 1947, and the 6-Day Sample from which they are drawn. The numbers in parentheses are Terman–Merrill Form L Binet IQ scores. No IQ scores are given for cells with fewer than five participants.

The participants are surviving members of the 6-Day Sample of the Scottish Mental Survey of 1947; they were all born in 1936 (Deary, Whalley, & Starr, 2009; MacPherson, 1958; Maxwell, 1969; Scottish Council for Research in Education, 1949). On the 4th of June 1947 the Scottish Council for Research in Education (SCRE) attempted to test every child born in 1936 and attending school in Scotland on the Moray House Test No. 12. They tested 70,805 of a possible 75,252 (Fig. 1). The children born on the first day of the even-numbered months were chosen to form a population-representative sample of the 1936-born population living in Scotland at a mean age of 11 years (MacPherson, 1958). In 1947, they recruited 1208 individuals from a possible 1215. The participants in the 6-Day sample were followed up on a further 14 occasions between 1947 and 1963, by which time they were 27 years old. Data on intelligence, personality, education, health, occupations, and family were gathered. Near to the time of the Scottish Mental Survey 1947 there was a 25-item Sociological Schedule, predominantly completed by head-teachers and school nurses (Scottish Council for Research in Education, 1949, pp. 27–48). Additional schedules were completed by head-teachers in 1950 and on leaving school to enter employment or further education. Participants or their families were then visited at home or, in some circumstances, requested to complete schedules themselves, on a further 13 occasions between 1951 and 1963. A special effort was made to contact 6-Day Sample members on the last follow-up in 1963, and SCRE obtained data from 1104 individuals (Maxwell, 1969, p. 20).

Previously, we conducted an anonymised linkage study on the 6-Day Sample, relating childhood data with death and health data (Deary, Batty, Pattie, & Gale, 2008). More recently, we obtained permission to conduct two types of new research with the 6-Day Sample, (1) to perform more up-to-date and comprehensive anonymised linkage between the data gathered between 1947 and 1963 and health and mortality data, and (2) to contact individuals in the 6-Day Sample via a third party (the National Health Service Central Register; NHSCR) in order to invite them to a follow-up study in older age. The present report is based on the latter part of the research programme. The process of obtaining permissions for the research programme is documented in a separate article (Brett & Deary, 2014). The study was approved by NHS Research Ethics Committee Scotland A. The NHSCR traced 1204 of the original 1208 based on the information provided from the 6-Day Sample files in Scottish Council for Research in Education (Fig. 1).

### 2.2 Measures

Data collected between 11 and 27 years of age (i.e., up to 1963).

#### 2.2.1 Sex

Male or female gender was recorded on the Scottish Mental Survey 1947 ledgers and in the Sociological Schedule.

#### 2.2.2 Family size

Number of children in the proband's family was recorded on the Scottish Mental Survey 1947 ledgers and in the Sociological Schedule.

#### 2.2.3 Father's occupational social class

Father's job was recorded in the Sociological Schedule, which gave some detailed guidance on how to record the occupation. It was coded into 6 social classes using the UK's 1951 Classification of Occupations (General Register Office, 1956; Knight, 1967).

#### 2.2.4 Childhood home occupancy

The Sociological Schedule contained information on the number of ‘apartments’ (including bedrooms, public rooms and the kitchen, but not bathrooms) in the home and the number of individuals in the home. The latter was divided by the former to obtain home occupancy.

#### 2.2.5 Height

This was recorded in the Sociological Schedule, which gave some details on how it should be recorded.

#### 2.2.6 Secondary school denomination

This was coded as non-denominational or Roman Catholic (see Paterson, Calvin, & Deary, in press).

#### 2.2.7 Personality

One of the follow-ups of the 6-Day Sample study was a questionnaire that took place in 1950, when the children were about 14 years old. It was completed by teachers. The following six personality characteristics were rated by the teachers on a 5-point scale: “conscientiousness”, “perseverance”, “stability of moods”, “desire to excel”, “originality”, and “self-confidence”. This was described in Maxwell (1969, p. 50) and Deary et al. (2008). The six items were subjected to principal components analysis, which suggested there were two components. These were rotated, and the scores on the first rotated component were saved. These scores were then residualised on the Terman–Merrill Binet test IQ scores. The correlations between the IQ-adjusted component scores and the individual items were “conscientiousness” = .87, “perseverance,” = .78, “stability of moods” = .70, “desire to excel” = .60, “originality” = .25, and “self-confidence” = − .05. We named the component ‘dependability’.

#### 2.2.8 3- or 5-year educational plan

The follow-up schedules of the 6-Day Sample recorded whether the proband had been allocated to a 3- or 5-year educational course at secondary school (Paterson, Pattie, & Deary, 2010). Three years corresponded to leaving school at the minimum permitted age of 15 years. Some who left school on attaining their 15th birthday did not complete the full three years of secondary schooling. The 5-year courses were intended to lead to the Leaving Certificate and thence to university or the professions.

#### 2.2.9 Educational attainment to age 27 years

Based on all the information from the follow-up schedules of the 6-Day Sample—including education obtained at school and later, up to age 27—a rating was made for each person of their highest educational qualifications. A five-grade rating was used, from ‘none’ to ‘higher professional or degree’. For more information see Paterson et al. (2010).

#### 2.2.10 Proband's own occupational social class at 27 years

Information on this was derived from the follow-up schedules of the 6-Day Sample. Where there was more than one occupation, the highest-level occupation was used. This was coded into 6 social classes using the UK's classification of occupations (General Register Office, 1956; Knight, 1967).

#### 2.2.11 IQ from Terman–Merrill Binet Test

The Form L of the Terman–Merrill revision of the Binet Test was applied individually to all members of the 6-Day Sample. This was available from the 6-Day Sample records. The testing was done by more than 25 different psychologists and students (Scottish Council for Research in Education, 1949, pp. 50).

#### 2.2.12 IQ from Moray House Test No. 12 (MHT)

Scores on the MHT were obtained from the Scottish Council for Research in Education records of the full Scottish Mental Survey 1947. This is a group-administered test with a 45-minute time limit. It has a predominance of verbal reasoning items. It also has some numerical and other items. The maximum score is 76. It was converted to an IQ-type score (mean = 100, SD = 15) based on the sample, after correcting for age at the time of the Scottish Mental Survey 1947.

#### 2.2.13 Cognitive testing in older age

All cognitive testing in older age was done by telephone by a single tester (CEB). The materials for the testing were sent by post prior to the interview. Inside the envelope that was sent to the participants was another envelope with the testing materials. This was securely sealed and was clearly marked as not to be opened until instructed at the time of the telephone interview. At that time, the interviewer led the participant through each test, in the following, fixed order.

#### 2.2.14 Mini-Mental State Examination (MMSE; Roccaforte, Burke, Bayer, & Wengel, 1992)

This telephone-administered version of the Mini-Mental State for telephone administration was used as a screening test for possible pathological cognitive decline. The maximum possible score was 26.

#### 2.2.15 National Adult Reading Test (NART; Nelson & Willison, 1991)

The participant was sent a single A4 sheet with the 50 NART words printed in capitals in two columns. They were asked to read the words. The NART's words are irregular in grapheme-phoneme association and/or stress. Therefore, pronunciation is likely to be incorrect if people do not already know the words. The number of correct pronunciations was used as the score.

#### 2.2.16 Semantic Fluency (Lezak, Howieson, & Loring, 2004)

This is often used as a test of executive function. The participant was asked to name as many animals as they could think of in 1 min. Prior to that, a short practice was given using clothing as the category.

#### 2.2.17 Rey Auditory Verbal Learning Test (AVLT; Lezak et al., 2004)

This is a test of verbal declarative memory. The participant read a list of 15 short, unconnected words. They were asked to repeat as many as they could recall. The word list was repeated four more times, with a recall after each. A second list was then read, with immediate recall. There was then a recall of the first list, without its being read again. There was then a further recall of the first list after a gap in which other tests took place.

#### 2.2.18 Symbol Digit Modalities Test, oral version (Smith, 1982)

This is a test of processing speed. For this test, the participant was sent a single sheet of US letter-size paper. This was contained inside a cardboard folder in the sealed test envelope so that it was not seen until the testing began. At the top of the sheet was a ‘key’ which has a row of nine symbols. Below each symbol is a number. Below that are 8 rows, each containing 15 symbols, with empty boxes below them where the participant is asked to put the correct number. The first 10 are for practice. In this test, the participant was asked to call out the correct number. The score was the number of correct items completed in 90 s.

#### 2.2.19 Serial 7 s

In this test, the participant was asked to count backwards from 100 in 7 s as quickly as possible. Scores on this test were not used in this report, because there was variance in how many errors were made, and there is no clear scoring method to adjust the time taken to complete the test to take into account the number of errors made.

#### 2.2.20 Raven's Standard Progressive Matrices (Raven, 1998)

This is a test of non-verbal reasoning. It has 60 items, in 5 sets. Each item has a pattern or matrix with an element missing. The participant must choose between the answer options which one correctly completes the pattern or matrix. The participant was guided on how to begin the test. They then stated the correct items over the telephone to the tester. The time limit for completion was 20 min. The participants sent the Raven's booklet and other testing materials back to the research team by post in a stamped, addressed envelope that was provided.

## 3 Results

Of the original 1208 members of the 6-Day Sample who took the Terman–Merrill IQ test in 1947, 635 were traced as being alive and living in Great Britain in 2013 (Fig. 1). They were invited to take part in our follow-up study. Of these, 172 completed a questionnaire and 131 of these took part in the telephone interview. Data from the 131 are the focus of this report. The descriptive data from childhood and young adulthood that were related to cognitive scores from older age are shown in Table 1, for the whole 6-Day Sample, for those alive and living in Great Britain (Scotland, England, and Wales) in older age, and for those who provided cognitive data in the telephone interview in older age. Those who provided cognitive data in older age were, on average, rated as being higher on the personality trait of dependability by teachers when young, were more likely to have a 5-year educational plan, had higher educational attainments, had higher occupational social class at 27 years, and had higher mean Terman–Merrill and MHT IQ scores at age 11.

The cognitive, social and educational data from childhood and young adulthood were tested for associations with cognitive test scores in older age. The NART is used to estimate prior intelligence, so it was considered separately from the others, which were intended to be assessments of current cognitive abilities. These latter five tests were considered on their own, and were also combined into a ‘fluid general intelligence’ component. This was formed using principal components analysis. Only one component had an eigenvalue of > 1, and the scree slope showed one component accounting for 52.4% of the total variance, on which all tests loaded highly: Mini-Mental State Examination = .73, Raven's Standard Progressive Matrices = .75, Symbol Digit Modalities Test = .83, Rey Auditory Verbal Learning Test = .69, Semantic Fluency = .60.

The correlation between the NART at age 77 and the Terman–Merrill at age 11 was .72, and between NART and the Moray House Test at age 11 was .66 (Table 2). NART correlated highly (≥ .5) with educational attainment, occupational social class at age 27, and the length of the school course. There were smaller, significant correlations indicating that people with lower NART scores had more deprived social backgrounds and were smaller in height. We note the many correlations performed in Table 2, and that the p values are not adjusted for multiple testing, and we urge special caution in interpreting those associations that have p values greater than about .01; however, we also note that the principal outcomes of interest were the NART and fluid general intelligence, and that the cognitive outcomes are strongly correlated and do not, therefore, involve independent tests. The correlations with fluid general intelligence followed the same pattern, but were lower. The correlation of fluid general intelligence with the Terman–Merill was .50 and with the Moray House Test was .52. Of the individual tests that made up the fluid intelligence component, Raven's Standard Progressive Matrices and the Symbol Digit Modalities Test had the highest correlations with the childhood intelligence measures. In some cases the Raven correlations were higher than those of the fluid intelligence component. Raven correlated .55 with the Terman–Merrill and .49 with the Moray House test, and .47 with educational attainment. Despite the brevity and relative simplicity of the Symbol Digit Modalities Test, it had sizeable correlations with the cognitive, educational and social class variables of the person from childhood and youth.

The next analysis asked which variables from childhood and youth together best predicted cognitive test scores in older age (Table 3). Stepwise linear regression was used. Only those with significant bivariate associations were considered, and the criterion for entry was p < .05. For the NART, the R was .81 and the adjusted R square was .643 with significant positive contributions from the Terman–Merrill, Moray House Test, length of educational course, and educational attainment. For fluid general intelligence the R was .57 and the adjusted R square was .31, with positive contributions from the Terman–Merrill and the length of the educational course. For Raven, only the Terman–Merrill was significant, so the estimate was the same as the bivariate correlation.

The next analysis asked how well NART retrodicted the two childhood intelligence test scores, and whether educational attainment and occupational social class information could improve upon that (Table 4). For the Terman–Merrill the R was .75 and the adjusted R square was .55, with the major contribution coming from the NART and a small positive contribution from educational attainment. For the Moray House Test, only the NART was significant, with an R of .66 and an adjusted R square of .43.

## 4 Discussion

This is the first of the long-term follow-up studies of the life-course stability of intelligence differences based on the Scottish mental surveys that has had individually-administered IQ test data from childhood (compare those summarised in Deary, 2014). Previous studies had group-administered tests. The correlation (r) of .72 between the Terman–Merrill IQ at age 11 and the National Adult Reading Test at age 77 years is the highest raw correlation in these childhood-to-older-age follow-up studies. The addition of the Moray House Test and educational information took the R to greater than .8 across the 66-year period. These associations were higher than those for the fluid general intelligence component and for Raven's Standard Progressive Matrices. Education, as well as childhood cognitive ability, shows strong correlations between childhood and cognitive test scores in older age. When the study turned from predicting older age cognitive ability to retrodicting prior intelligence from the NART, an R of .75 was obtained, in which educational attainment added a little information beyond the NART. The retrodiction was higher for the Terman–Merrill IQ than for the Moray House Test. None of the estimates has been adjusted for test reliability or the cognitive ability-based restriction of range in the sample, so they will be under-estimates of the true values.

Strengths of the study are the 66-year follow-up period, the narrow age range of the participants, the fact that the original sample was population-representative, the almost-complete tracing from youth to those alive in older age, there being individually-administered and group-administered valid IQ-type tests in childhood, the rich social, educational and occupational data from childhood and young adulthood, and the extensive cognitive testing in older age. Limitations of the study are the relatively small numbers tested at age 77, the cognitive testing being done via telephone interview rather than face-to-face, the small percentage of those contacted in older age who took part and provided data, and that there was not a replication sample in which to test the robustness of the regression models. The selection factors on the follow-up of participants may be seen in part in Table 1 and Fig. 1. Those who were alive in older age and those who emigrated were higher in mean childhood IQ. Down the line from those factors, the sample who returned the questionnaire had higher IQ scores than those who did not, and the sample who took part in the telephone interview had the highest mean childhood IQ of any group. We note that, although the correlations obtained here between cognitive tests administered 66 years apart are the highest we have reported, the numbers tested are not high enough to make these correlations significantly greater than those reported previously (Deary, 2014; Dykiert & Deary, 2013). We note that it is a happenstance that the cognitive data were available from age 11 in the present study. However, a recent meta-analysis of the continuity of cognitive ability across the life course found that the rank-order stability of intelligence is close to reaching its asymptote at age 11 (Tucker-Drob & Briley, 2014). This means that the present study is not much disadvantaged by having the original test scores from age 11 rather than in young adulthood.

Given that the Terman–Merrill IQ from age 11 had a high correlation on its own with NART at age 77, it is interesting to find that an additional age-11 IQ-type test (MHT) and two education-related variables added significantly more variance (Table 3). The first thing this tells us is that any test—even an omnibus one such as the revision of the Binet test—will not capture all relevant cognitive variance. The MHT has a preponderance of verbal reasoning items (Deary, Whalley, & Starr, 2009; Scottish Council for Research in Education, 1933), and it might have picked up some relevant verbal variance in the NART that the Terman–Merrill Binet test did not. Whereas the R square change for the MHT was less than 2% of the variance, there was a larger contribution from whether the child was on a 3- or 5-year secondary school course. The choosing of children for the longer course reflects the real-world process of selection, which was based on a mixture of tests of intelligence, tests of attainment (including English), teacher judgements, and parental wishes. The 5-year course would involve more foreign-language teaching. Therefore, it is perhaps not surprising that the various contributors to the course allocation might pick up information relevant to later vocabulary that was in addition to general mental ability. Similarly, beyond the two IQ-type tests and the 3- or 5-year course allocation, the best qualification level attained up to age 27 added almost 3% of the variance in addition. This might be a two-way association. First, extra education is likely to add to verbal knowledge. Second, it is also possible that having more verbal ability—not fully picked up by the other two IQ tests and the school course allocation—could lead to people acquiring more education either because verbally-able people are drawn to and choose to have more education, or because they are more likely to be selected.

The NART is the epitome of crystallised cognitive tests, holding its mean level well with age and even in cases of mild dementia (Dykiert & Deary, 2013; McGurn et al., 2004). Therefore, it might be expected that education and other enculturation might have a particular predictive power on it and other verbal tests (Ritchie et al., 2013). When we formed a fluid cognitive ability component score at age 77—based on tests assessing cognitive domains more prone to negative effects of ageing—we again found that having been on a 3- or 5-year education course made a sizeable additional predictive contribution on top of the Terman Merrill IQ score at age 11. There was no additional contribution from the MHT or educational qualifications. The fluid cognitive component contained some tests with verbal content, such as Semantic Fluency, Rey Auditory Verbal Learning Test, and Mini-Mental State Examination. Therefore, the regression model was run again with Raven's Matrices, an entirely non-verbal test. For Raven's scores, the only predictor from youth was the Terman–Merrill IQ score, with no additional predictive power gained by the MHT, educational course, or educational qualifications. This contributes support to our previous suggestion that education might contribute more to the products of intelligence (crystallised abilities) than its process or mechanism (fluid abilities) (Ritchie et al., 2013).

Before the NART and similar tests were available, education was one variable that was used as a proxy for prior cognitive ability (Wilson et al., 1978), and adding demographic variables (e.g., occupational social class) to the NART has been found to increase slightly its power to estimate cognitive ability (Crawford, Nelson, Blackmore, Cochrane, & Allan, 1990). Here, we found that adding educational qualifications to NART provides a little extra retrodictive power in estimating the Terman–Merrill IQ score from age 11, though not the MHT at the same age. Because NART is intended to estimate peak prior intelligence, and that the peak will have occurred after age 11, it might be that educational qualifications are adding some power—in the case of the Terman–Merill scores—to obtain some of the change in individual cognitive differences between age 11 and young adulthood.

In conclusion, this is, as far as we are aware, the longest follow-up study of intelligence that has used individually-assessed cognitive ability test scores in childhood and older age. The correlation between the Binet IQ at age 11 and verbal ability at age 77 was .72. Adding on a group-administered test at age 11 and educational information raised the multiple R to > .8. The retrodiction of childhood Binet IQ from NART at age 77 was also strong, and slightly enhanced by education to achieve an R ~ .75. Though there was strong participant selection between childhood and older age, the study benefitted from being based on a representative sample at baseline, and having detailed information on the selection process thereafter.

## Figures and Tables

**Table 1:** Descriptive data from childhood and young adulthood for the whole 6-Day Sample (N = 1208), for those alive in 2013 and invited to follow-up (n = 635), and those who provided cognitive data at telephone interview (n = 131^a^).

		Whole 6-Day Sample	Invited to follow-up	Provided cognitive data at follow-up
Sex (N, %)	Male	590 (48.8)	265 (41.7)	59 (45)
	Female	618 (51.2)	370 (58.3)	72 (55)
Family size (M, SD)		3.8 (2.4)	3.6 (2.2)	3.0 (1.6)
		N = 1204	N = 632	N = 130
Father's occupational social class (N, %)	Professional	31 (2.6)	17 (2.7)	8 (6.1)
	Intermediate	119 (9.9)	82 (12.9)	22 (16.8)
	Skilled	629 (52.1)	329 (51.8)	68 (51.9)
	Semi-skilled	203 (16.8)	96 (15.1)	16 (12.2)
	Unskilled	206 (17.1)	94 (14.8)	14 (10.7)
	No information	20 (1.7)	17 (2.7)	3 (2.3)
Childhood home occupancy (M, SD)		2.1 (1.1)	2.0 (1.1)	1.7 (1.0)
		N = 1155	N = 601	N = 118
Height (inches; adjusted for age) (M, SD)		54.0 (2.8)	54.1 (2.8)	54.6 (2.8)
		N = 1148	N = 595	N = 116
Secondary school denomination (N, %)	Non-denominational	987 (81.7)	522 (82.2)	114 (87.0)
	Roman Catholic	221 (18.3)	113 (17.8)	17 (13.0)
Teacher rating of dependability (M, SD)		0.00 (1.00)	0.09 (0.97)	0.24 (0.97)
		N = 1191	N = 628	N = 130
Does pupil have a 5-year plan? (N, %)	No	847 (70.1)	417 (65.7)	49 (37.4)
	Yes	351 (29.1)	215 (33.9)	82 (62.6)
	Missing	10 (0.8)	3 (0.5)	0 (0)
Educational attainment to age 27 years (N, %)	None or low	682 (56.5)	343 (54.0)	37 (28.2)
	Trade, etc.	309 (25.6)	153 (24.1)	41 (31.3)
	City & Guilds, ONC, HNC	82 (6.8)	43 (6.8)	8 (6.1)
	Nursing, non-graduate teaching	60 (5.0)	40 (6.3)	16 (12.2)
	Higher professional, degree	72 (6.0)	53 (8.3)	29 (22.1)
	Missing	3 (0.2)	3 (0.5)	0 (0)
Proband's own social class at age 27 years (N, %)	Professional	46 (3.8)	31 (4.9)	14 (10.7)
	Intermediate	164 (13.6)	105 (16.5)	38 (29.0)
	Skilled	696 (57.6)	348 (54.8)	63 (48.1)
	Semi-skilled	179 (14.8)	99 (15.6)	13 (9.9)
	Unskilled	111 (9.2)	47 (7.4)	2 (1.5)
	Missing	12 (1.0)	5 (0.8)	1 (0.8)
IQ from Terman–Merrill Binet test (M, SD)		102.5 (20.1)	104.7 (20.6)	118.5 (19.1)
		N = 1208	N = 635	N = 131
IQ from Moray House Test No. 12^b^ (M, SD)		100.0 (15.0)	101.9 (14.6)	111.2 (10.8)
		N = 1112	N = 594	N = 125

a This is the maximum N; some cell sizes are smaller, because of missing data.

b This was computed by using linear regression to adjust the raw Moray House Test No. 12 score (out of 76) from the Scottish Mental Survey 1947 for age, saving the standardised residuals, multiplying the result by 15, and adding 100.

**Table 2:** Pearson^a^ correlations (p values) in the 6-Day Sample follow-up group between cognitive ability test scores in older age and social, educational, occupational, and cognitive variables from childhood and young adulthood.

	National Adult Reading Test	Fluid general intelligence^b^	Mini-Mental State examination	Raven's Standard Progressive Matrices	Symbol Digit Modalities Test	Rey Auditory Verbal Learning Test	Semantic Fluency
Gender^b^	− .15 (.087)	− .06 (.50)	.06 (.53)	− .16 (.073)	.01 (.92)	.18 (.045)	− .24 (.005)
Size of family	− .14 (.12)	− .21 (.021)	− .11 (.23)	− .18 (.040)	− .13 (.16)	− .10 (.24)	− .10 (.28)
Father's occupational social class	− .17 (.054)	− .08 (.36)	.09 (.29)	− .13 (.14)	− .11 (.23)	− .01 (.88)	− .09 (.31)
Home occupancy rate in childhood	− .26 (.004)	− .27 (.004)	− .19 (.041)	− .19 (.039)	− .23 (.015)	− .21 (.026)	− .18 (.054)
Teacher's rating of dependability	− .05 (.53)	− .10 (.29)	− .07 (.40)	− .02 (.78)	− .00 (.96)	− .08 (.37)	− .06 (.51)
3- or 5-year school course^c^	.58 (< .001)	.47 (< .001)	.23 (.009)	.39 (< .001)	.45 (< .001)	.27 (.002)	.25 (.004)
Secondary school denomination^c^	− .04 (.67)	− .10 (.25)	− .00 (.98)	− .09 (.33)	− .03 (.73)	− .07 (.41)	− .12 (.17)
Educational qualification level to age 27	.62 (< .001)	.40 (< .001)	.18 (.036)	.47 (< .001)	.32 (< .001)	.15 (.082)	.19 (.027)
Social class at age 27	− .50 (< .001)	− .32 (< .001)	− .12 (.15)	− .32 (< .001)	− .32 (< .001)	− .12 (.19)	− .21 (.017)
Height in childhood	.19 (.041)	.17 (.081)	.20 (.035)	.23 (.011)	.15 (.11)	.09 (.34)	.02 (.82)
Terman–Merrill Binet IQ score	.72 (< .001)	.50 (< .001)	.33 (< .001)	.55 (< .001)	.42 (< .001)	.26 (.003)	.25 (.004)
Moray House Test IQ score	.66 (< .001)	.52 (< .001)	.28 (.002)	.49 (< .001)	.49 (< .001)	.36 (< .001)	.26 (.003)
Mean (SD)	34.9 (8.0)		24.7 (1.8)	33.6 (7.6)	42.6 (10.1)	46.5 (11.8)	18.5 (5.2)
N	131		131	130	129	128	131

a Spearman correlations were similar, with small differences at the second decimal place.

b Based on a principal components analysis of all the tests to the right of this column.

c Point-biserial correlation. Ns for correlations vary, depending on Ns for cognitive testing in older age (see above) and Ns for data from youth (see Table 1).

**Table 3:** Stepwise multiple linear regression with cognitive ability at age 77 years as the outcome and childhood and young adulthood cognitive, educational, social and occupational variables as predictors.

		National Adult Reading Test	Fluid general intelligence	Raven's Standard Progressive Matrices
N with full data		112	107	111
R		.810	.572	.552
Adjusted R square		.643	.315	.298
R square change (standardised beta)	Terman–Merrill Binet IQ score	.547 (.317)	.266 (.331)	.305 (.552)
	Moray House Test IQ score	.017 (.209)	–	–
	3- or 5-year school course	.062 (.198)	.062 (.310)	–
	Educational qualification level to age 27	.029 (.251)	–	–
	Home occupancy rate in childhood	–	–	–
	Social class at age 27	–	–	–

**Table 4:** Stepwise multiple linear regression with childhood (prior) cognitive ability as the ‘outcome’ and National Adult Reading Test scores at age 77 and young adult educational and occupational variables as predictors.

		Terman–Merrill Binet IQ score	Moray House Test IQ score
N with full data		130	124
R		.746	.662
Adjusted R square		.550	.434
R square change (standardised beta)	National Adult Reading Test	.525 (.582)	.438 (.662)
	Educational qualification level to age 27	.032 (.229)	–
	Social class at age 27	–	–
